# Expanding the dermoscopic spectrum of Merkel cell carcinoma: A case report

**DOI:** 10.1016/j.jdcr.2026.05.008

**Published:** 2026-05-12

**Authors:** Corrine Hutchinson, Ophelia Pilkinton, Jason Eakes, Allison Jones

**Affiliations:** a UTHSC College of Medicine, University of Tennessee, Memphis, Tennessee; b Kaplan-Amonette Department of Dermatology, UTHSC College of Medicine, University of Tennessee, Memphis, Tennessee

**Keywords:** dermoscopy, Merkel cell carcinoma, neuroendocrine carcinoma, non-melanoma skin cancer, surgery, vascular lesion

## Introduction

Merkel cell carcinoma (MCC) is a rare, aggressive cutaneous malignancy that classically affects older patients with lightly pigmented skin. It typically presents as a rapidly growing, painless, red-violet or, less commonly, skin-colored, or blue-red nodule on sun-exposed areas, particularly the head-neck region and the distal extremities.^1^ The incidence of MCC is significantly increased in immunosuppressed patients, including solid organ transplant recipients. Early recognition is critical, as MCC carries a high risk of local recurrence, nodal involvement, and distant metastasis.^2^

Dermoscopic evaluation of MCC typically demonstrates a milky red to pink background, polymorphous vascular patterns, including linear irregular, arborizing, glomerular, and dotted vessels, as well as white structureless areas with a notable absence of pigmentation.^1^, ^2^, ^3^, ^4^, ^5^, ^6^ However, dermoscopic findings may be variable and remain incompletely characterized.^1^ Because MCC lacks specific clinical and dermoscopic features, it is frequently misdiagnosed as other benign or malignant cutaneous lesions which may delay appropriate diagnosis and treatment.^2^

Here, we report a case of MCC in an immunosuppressed patient presenting with striking jewel-like dark-red and purple lacunae, an atypical feature not previously documented. This case expands the known dermoscopic spectrum of MCC and underscores the importance of prompt biopsy of atypical vascular lesions.

## Case report

**Fig 1:**
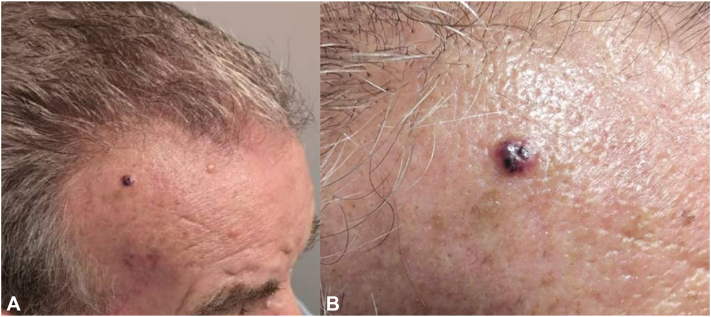
Solitary papule on the right forehead. **A,** Solitary red to violaceous papule on the right forehead. **B,** Magnified view of papule on right forehead.

A 67-year-old male with a history of melanoma in situ, dysplastic nevi, multiple non-melanoma skin cancers, and prior liver transplantation on chronic tacrolimus immunosuppression presented with a 2 to 3 week history of a spontaneously appearing lesion on the right forehead. Physical examination revealed a solitary, well-circumscribed, red and purple papule measuring approximately 0.5 × 0.5 cm (Fig 1).

**Fig 2:**
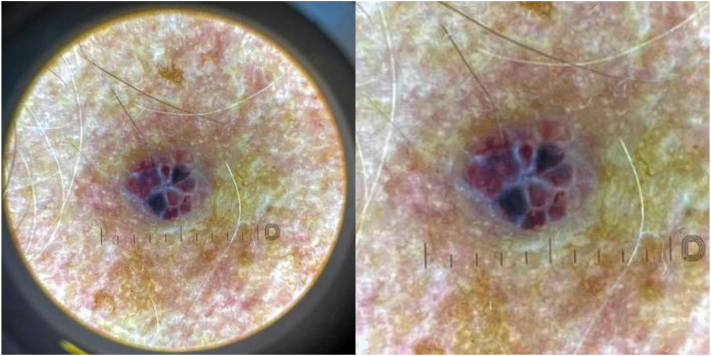
Dermoscopy of lesion.

Dermoscopic evaluation demonstrated striking jewel-like clustered dark red-to-violaceous lacunae (well-demarcated, round-to-oval vascular spaces). Due to the unusual dermoscopic vascular pattern, a tangential biopsy was obtained (Fig 2). Histopathologic examination with immunohistochemistry revealed tumor cells positive for CD56 and synaptophysin, with focal positivity for cytokeratin 20, and negative staining for BerEP4 and thyroid transcription factor 1, confirming the diagnosis of Merkel cell carcinoma.

The patient underwent staging with PET-CT, which showed no evidence of distant metastatic disease. Sentinel lymph node mapping with SPECT-CT localized 3 right-sided sentinel nodes, including 2 preauricular nodes and 1 level II cervical node. The patient subsequently underwent wide local excision with sentinel lymph node mapping and biopsy, followed by split-thickness skin graft placement. Postoperatively, referral for adjuvant radiation therapy was recommended.

## Discussion

Merkel cell carcinoma (MCC) is an aggressive cutaneous neuroendocrine carcinoma with increased incidence and high mortality in immunosuppressed patients, including solid organ transplant recipients.^2^ In this case, the patient’s history of liver transplantation and chronic tacrolimus therapy represented a major predisposing risk factor. Given that MCC frequently lacks distinctive clinical features and may mimic benign vascular proliferations, dermoscopic characterization is particularly valuable for improving early recognition and prompting timely biopsy.

Previously published dermoscopic descriptions of MCC consistently emphasize amelanotic, vascular-predominant lesions, most commonly demonstrating a milky red background with polymorphous vessels, particularly linear irregular patterns.^1^, ^2^, ^3^, ^4^, ^5^, ^6^ In contrast, the lesion described in this report lacked a classic milky red structureless background and did not display clearly polymorphous or linear irregular vessels. Instead, dermoscopy revealed densely clustered dark red–purple lacunae with organized septation. This pattern likely corresponds to blood-filled spaces and intratumoral hemorrhage separated by tumor nests and stromal septa rather than true fibrosis, given the short clinical duration. Vazmitel et al in 2008 found that 20% of MCC cases were found to have foci of prominent vascular changes on histology, including a peliosis-like pattern characterized by blood-filled sinusoidal spaces. This could plausibly account for the lacunar-appearing structures on dermoscopy in this case, although this correlation has not been previously described.^7^ This finding further illustrates the variability of MCC dermoscopy and suggests that vascular architecture may manifest in patterns beyond those most frequently described.

This morphology has practical diagnostic implications. Small, well-circumscribed red papules on sun-exposed skin are often clinically attributed to benign vascular lesions. Cherry angiomas typically demonstrate well-demarcated red, maroon, or blue lacunae separated by pale septa, whereas pyogenic granulomas show homogeneous red structureless areas with white rail lines and/or a peripheral collarette.^8^ The lesion in this case, although visually vascular, did not conform to classic benign dermoscopic patterns. Failure to recognize this distinction could result in misclassification as a benign hemangiomatous proliferation and delay diagnostic biopsy.

Several limitations warrant acknowledgment. This report represents a single case and does not establish the frequency or reproducibility of this vascular morphology in MCC. Dermoscopic terminology remains descriptive rather than standardized, and the phrase “jewel-like vascular lacunae” may be subject to interpretive variability. Image acquisition factors, including lighting and magnification, may also influence perceived color intensity and vascular configuration. Additionally, published dermoscopic data on MCC are derived primarily from small case series, limiting the robustness of comparative analysis. Although histopathologic and immunohistochemical findings confirmed the diagnosis, the tangential biopsy specimen limited assessment of the lesion’s full architectural features. Despite these limitations, this case adds to the evolving dermoscopic spectrum of MCC by documenting a vascular configuration not previously emphasized in peer-reviewed reports. Recognition that MCC may present with compact vascular structures broadens the range of concerning vascular patterns and supports maintaining diagnostic vigilance when evaluating new vascular-appearing lesions, particularly in high-risk patients.

## Conclusion

This case describes Merkel cell carcinoma presenting with distinctive densely clustered dark red and purple vascular lacunae on dermoscopy, expanding the range of vascular patterns reported in MCC. In contrast to the more commonly described milky red background with polymorphous or linear irregular vessels, this lesion demonstrated compact, bundle-like vascular structures that could clinically resemble a benign vascular proliferation. Recognition that MCC may present with atypical vascular-dominant dermoscopic patterns is particularly important in immunosuppressed patients, in whom a low threshold for biopsy of new vascular lesions may reduce diagnostic delay and facilitate earlier detection of this aggressive malignancy.

### Declaration of generative AI and AI-assisted technologies in the writing process

None.

## Conflicts of interest

None disclosed.
